# *Brocchia cinerea* (Delile) Vis. Essential Oil Antimicrobial Activity and Crop Protection against Cowpea Weevil *Callosobruchus maculatus* (Fab.)

**DOI:** 10.3390/plants11050583

**Published:** 2022-02-22

**Authors:** Abdelkrim Agour, Ibrahim Mssillou, Hamza Mechchate, Imane Es-safi, Aimad Allali, Azeddin El Barnossi, Omkulthom Al Kamaly, Samar Zuhair Alshawwa, Abdelfattah El Moussaoui, Amina Bari, Badiaa Lyoussi, Elhoussine Derwich

**Affiliations:** 1 Laboratory of Natural Substances, Pharmacology, Environment, Modeling, Health, and Quality of Life, Faculty of Sciences Dhar El Mahraz, University Sidi Mohamed Ben Abdellah, Fez 30050, Morocco; abdelkrimagour1@gmail.com (A.A.); mssillouibrahim@gmail.com (I.M.); lyoussi@gmail.com (B.L.); elhoussinederwich@yahoo.fr (E.D.); 2 Laboratory of Inorganic Chemistry, Department of Chemistry, University of Helsinki, P.O. Box 55, FI-00014 Helsinki, Finland; Imane.essafi1@usmba.ac.ma; 3 Laboratory of Animal and Plant Production, Agro-Industry, Faculty of Sciences, Ibn Tofail University, P.O. Box 133, Kenitra 14000, Morocco; aimad.allali@uit.ac.ma; 4 Laboratory of Biotechnology, Environment, Agrifood, and Health, Faculty of Sciences Dhar El Mahraz, University of Sidi Mohamed Ben Abdellah, Fez 30050, Morocco; Azeddin.elbarnossi@usmba.ac.ma (A.E.B.); Abdelfattah.elmoussaoui@usmba.ac.ma (A.E.M.); aminabari3@gmail.com (A.B.); 5 Department of Pharmaceutical Sciences, College of Pharmacy, Princess Nourah Bint Abdulrahman University, P.O. Box 84428, Riyadh 11671, Saudi Arabia; SZAlshawwa@pnu.edu.sa

**Keywords:** *Brocchia cinerea*, essential oil, antimicrobial activity, *Callosobruchus maculatus*, pest control, insecticidal, cowpea

## Abstract

Antibiotics and synthetic pesticides are now playing a role in the spread of resistant pathogens. They continue to have negative consequences for animal and plant health. The goal of this work is to identify the chemical composition of *Brocchia cinerea* (Delile) Vis. essential oil (EO) using GC-MS(Gas Chromatography-Mass Spectrometer), evaluate its antimicrobial properties, and investigate its insecticidal and repellent effectiveness against *Callosobruchus maculatus* (*C. maculatus*). The GC-MS indicated the presence of 21 chemicals, with thujone (24.9%), lyratyl acetate (24.32%), camphor (13.55%), and 1,8-cineole (10.81%) being the most prominent. For the antimicrobial assay, the yeast *Candida albicans* was very sensitive to the EO with a growth inhibition diameter of (42.33 mm), followed by *Staphylococcus aureus* (31.33 mm). *Fusarium oxysporum* is the mycelia strain that appeared to be extremely sensitive to the utilized EO (88.44%) compared to the two species of Aspergillus (*A. flavus* (48.44%); *A. niger* (36.55%)). The results obtained in the microdilution method show that *Pseudomonas aeruginosa* was very sensitive to the EO, inhibited by a very low dose (0.0018 mg/mL). The minimum inhibitory concentration (MIC) results were between 0.0149 and 0.06 mg/mL. *B. cinerea* EO also demonstrated a potent insecticidal effect and a medium repulsive effect against *C. maculatus*. Thus, the LC_50_ value in the contact test was 0.61 μL/L of air, lower than that observed in the inhalation test (0.72 μL/L of air). The present study reveals that *B. cinerea* EO has the potential to be an antimicrobial and insecticidal agent with a better performance against several pathogenic microorganisms.

## 1. Introduction

Natural compounds found in herbal extracts and essential oils (EOs), as well as other secondary metabolites of plants, microbes, and enzymes, are gaining huge popularity nowadays, but they are still underutilized [1]. EOs have been shown in several studies to control the development of pathogens [2]. The overuse of chemical pesticides has had a negative effect on public health and the environment, and essential oils may be a viable alternative [3]. On the other side, bio-insecticides may be very effective, selective, and have little or no pest resistance and are non-toxic to the environment [4]. Insecticides made from plant EOs may offer safer and less environmentally damaging alternative control options [5]. *Brocchia cinerea* (Delile) Vis. (*B. cinerea*) is one of the plant species that belong to the Asteraceae family, known for their richness in essential oils. *B. cinerea* has traditional uses in the treatment of several diseases and infections related to bacteria [6,7,8], and due to its good preservative properties, additionally, it is used to filter goat butter [9]. *B. cinerea* has other biological activities: the powder of this species has an effect on the mycelial growth of *Penicillium italicum* (43.08%), the agent responsible for the blue mold of citrus fruits. 

Foods that have been stored are susceptible to postharvest loss in quality and quantity due to infestation by a variety of insect species.. The multivoltine pest *Callosobruchus maculatus* Fab. (Coleoptera: Bruchidae) is causing considerable harm to stored pulses in several tropical nations [10]. Bruchid beetles eat grains and make holes in them, which they utilize to lay eggs. Whole grain is often used, and these holes are often covered and filled with eggs [11]. The *C. maculatus* alone may cause up to 90% damage in a period of 3 to 6 months of storage [12]. The control technique is still reliant on fumigation and synthetic pesticides, both of which are hazardous to the environment. Pesticide residues endanger the health of living creatures and pose a serious threat to humans. In order to address rising resistance, the requirement for innovative and efficient bioactive insecticidal compounds has risen [11]. In order to address rising resistance, the requirement for innovative and efficient bioactive insecticidal compounds has risen [13]. Biopesticides derived from essential oils (EOs) seem to be a complementary or alternative method in crop production and integrated pest management aimed at mitigating the adverse effects of conventional synthetic pesticides [14]. On stored product insects, EOs had potentially toxic, repellant, and antifeedent effects [15].

The current study’s goal is to figure out the chemical composition by GC-MS of the essential oil of *B. cinerea*, harvested in the region of Tata (south-east of Morocco), to evaluate its antimicrobial power (against bacterial strains causing nosocomial affections) and antifungal effect (against agents of infection of food), and to carry out a first study on the insecticidal effect of the essential oils of *B. cinerea* against *C. maculatus*, one of most common pest species that attack various types of beans in storage [16].

## 2. Results and Discussion 

### 2.1. Extraction Yield and Phytochemical Analysis

The yield of essential oil from *B. cinerea* varies between 0.080 and 0.87% according to several studies [7]. However, the study of Guaouguaou et al. [17] showed that an extraction that lasted 6 h gave a yield of (0.92%). The yield obtained in our study was 0.39%. 

The essential oil’s GC-MS analysis revealed the presence of 21 components. The oil was distinguished by a significant concentration of oxygenated monoterpenes. (89.03%) and low content of hydrocarbon monoterpenes (10.64%). These results are in agreement with other studies: Chlif et al. [18] reported that the components identified in the dry aerial parts of *B. cinerea* were oxygenated monoterpenes (56.67%), monoterpene hydrocarbons (12.08%); in a different study, the components identified were oxygenated monoterpenes (95.40%) followed by monoterpene hydrocarbons (2.17%) [19]. In another research, the essential oil of this species was shown to contain oxygenated monoterpenes (82.3%) and monoterpene hydrocarbons (14.5%) [20].

**Figure 1:**
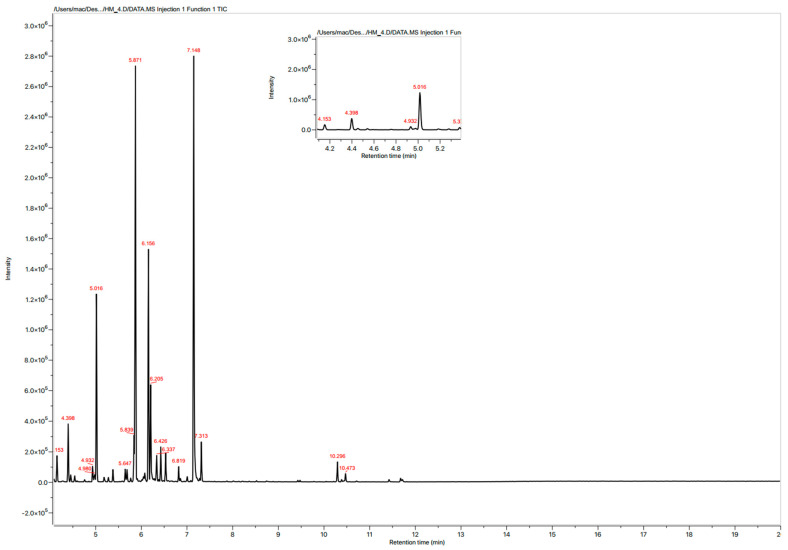
Chromatogram of *B. cinerea* essential oils GC-MS.

Twenty one constituents representing about 99.97% of the EO were identified (Figure 1 and Table 1), of which the major components were thujone (24.9%), lyratyl acetate (24.32%), camphor (13.55%), and 1,8-cineole (10.81%). Comparing these results with others reported in several studies (Table 2), it can be seen that the sample studied is distinct by its lyratyl acetate content, which represents almost a quarter of the totality. However, in the majority of cases, thujone or one of its isomers remain the major compounds, with the presence of santolina triene, 1,8-cineol, and camphor. The essential oil components of the plant might vary according on environment conditions, soil type and utilized portion, and harvest season.

### 2.2. Antimicrobial Activity

The findings of the EOs’ antibacterial and antifungal activities are provided in Table 3 and Table 4, respectively. *B. cinerea* EO exerted significant inhibitory activity against all microbial strains tested compared to synthetic antibiotics (streptomycin and fluconazole).

The yeast *C. albicans* was very sensitive to the EO with an inhibition diameter of (42.33 mm), followed by *S. aureus* (31.33 mm). In addition, the EOs showed lower zones of inhibition against *E. coli* and *B. subtills*, which were, respectively, 26.33 mm and 25 mm. However, *P. aeruginosa* showed a little resistance to the essential oil tested compared to the other bacterial species. The mycelia strain, which appears to be very sensitive to the oil used, is *F. oxysporum* (88.44%), compared to the two species of Aspergillus (*A. flavus* (48.44%); *A. niger* (36.55%)).

The results obtained in the microdilution method show that *P. aeruginosa* was very sensitive to *B. cinerea* EO, inhibited by a very low dose (0.0018 mg/mL). The concentration of 0.0037 mg/mL was sufficient to stop the growth of *E. coli*, while *B. subtilis* appeared to be the least sensitive with an inhibition starting from (0.037 mg/mL). Similarly, MICs between 0.0149 and 0.06 mg/mL were sufficient to arrest the growth of mycelia strains. In general, among all the strains studied, the two gram-negative bacteria (*E. coli* and *P. aeruginosa*) remained the most sensitive to essential oils extracted from *B. cinerea*. The present study reveals that essential oils extracted from the aerial parts of *B. cinerea* have the potential to be an antimicrobial agent against several pathogenic microorganisms.

Previous studies have been carried out on the antimicrobial effect of the essential oil of *B. cinerea*, or one of its major compounds, against strains belonging to the same genera as those examined in this study. Ghouti et al. [22] revealed that *B. cinerea* oil limits the development of *B. subtilis* by a minimal concentration of (MIC = 0.303 mg/mL). Similarly, the growth of *B. subtilis*, *E. coli*, and *S. aureus* was stopped at 1/500 *v*/*v* concentration [23]. In addition, Chouikh [24] found that the strains of *E. coli* and *S. aureus* showed great sensitivity to the concentrations (1/1, 1/2, 1/4, 1/8) of EO (flowering period) where the diameter of inhibition ranged between (21 mm to 50 mm), while *P. aeruginosa* showed stiff resistance with every concentration of essential oil.

The effect of the essential oil of *B. cinerea* also inhibits the growth of mycelial fungi. Reference [25] reported that the oil of this plant inhibits the growth of *A. niger* (50.80%) at a concentration of 1/250 *v*/*v*, but complete inhibition of its growth requires a concentration of 1/100 *v*/*v*. Similarly, Boussoula et al. [23] showed that oil stops the growth of *A. niger* at 1/250 *v*/*v*. It was shown in another study that the activity of essential oils of *B. cinerea* is high against a group of human pathogenic fungi, including *Candida albicans*, with minimum inhibitory concentrations of 3.2 to 4.7 mg/mL [26].

According to these observations, it can be speculated that the strong antimicrobial activity exerted by the EOs of *B. cinerea* can be attributed only to the major compounds (thujone, lyratyl acetate, camphor, and 1,8-cineole), which account for about 73.58% of its total composition, and it could be the result of synergies between the different constituents of the EO [27]. Thujone (major compound) or one of its isomers (A or B) have been reported as molecule inhibiting the growth of several microbial strains including (*S. aureus*, *P. aeruginosa*, *C. albicans, E. coli*, *A. niger*) [28,29,30,31]. 

The percentage of Lyratyl acetate in the studied essential oils is very significant (24.04%). Casiglia [32] showed that the essential oils of *Anthemis secundiramea*, containing (Z)-lyratyl acetate (14.6%) as a major compound, exhibits significant antibacterial action against a variety of pathogens, with very low MIC values (μg/ mL) (*B. subtilis* 12.5; *S. aureus* 25; *E. coli* 50; *P. aerogenosa* 100; *C. albicans* 50; *F. oxysporum* 12.5, and *A. niger* 6.25). Other EOs, containing (Z)-lyratyl acetate as the majority compound, inhibited the growth of *A. flavus* (74.4%) by a concentration of 20 μL/petri dish for 6 days) [33].

The antimicrobial activity of camphor depends on its synergistic action with 1,8-cineole or other compounds existing in EOs [34,35]. Silver nanoparticles produced greenly from camphor extract, on the other hand, demonstrated their potential to favorably suppress isolated bacterial pathogens of both American and European foulbrood [36].

1,8-cineole inhibited the growth of *A. niger* (52.3 ± 43%) and *F. oxysporum* (72.2 ± 2.6%) by a concentration of 0.918 mg/mL. In addition, its presence in the essential oil in large quantities generates a very remarkable antimicrobial power [37]. Wagner et al. [38], showed that the EOs of *Lavandula dentata*, containing eucalyptol (or 1,8-cineole) (34.33%), fenchone (17.78%), and camphor (15.75%), had significant fungicidal action against *Cercospora kikuchii*, *Cercospora sojina*, and *Septoria glycines* plant pathogenic fungi, with diameters of the inhibition zone of 34.00, 29.50, and 22.00 mm at a concentration of 5 μL/disc, respectively.

According to the results of later studies, it is assumed that the strong antimicrobial activity of the *B. cinerea* EOs is strongly due to the presence of lyratyl acetate, 1,8-cineole, and monoterpene alcohols (*p*-Mentha-1(7),8(10)-dien-9-ol(5.62%), 4-Terpineol (2.02%), (-) Borneol (1.52%); α-terpineol (1.67%)). Indeed, Dorman et al. [39] tested a large number of pure constituents of EOs against 25 different genera of bacteria and showed that thymol is the compound with the broadest spectrum of antibacterial activity followed by carvacrol and α-terpineol.

The EO’s antibacterial action was thought to include simultaneous cytomembrane breakdown, which resulted in the release of intracellular constituents including protein and K^+^ [40]. As for their mode of action, EOs cause cell wall damage by establishing a membrane potential across the cell wall and disrupting ATP assembly [41]. However, the inhibitory power of the molecules remains inferior to that exerted by EOs containing these molecules as majority compounds or in large quantities [40]; this boosted effect can be explained by the synergic effect of the molecules contained in the EO.

### 2.3. Insecticidal Activity

**Figure 2:**
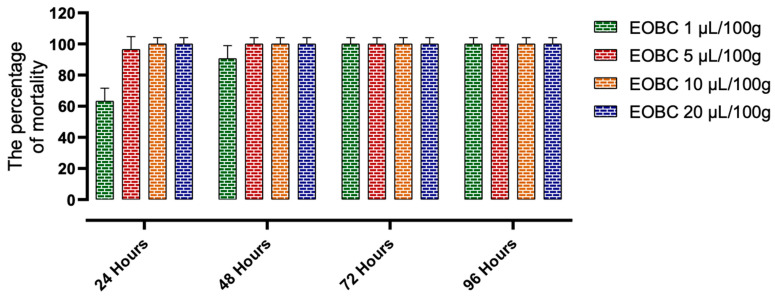
The effects of inhaling essential oil of *B. cinerea* (EOBC) on the mortality of adults of *C. maculatus*.

**Figure 3:**
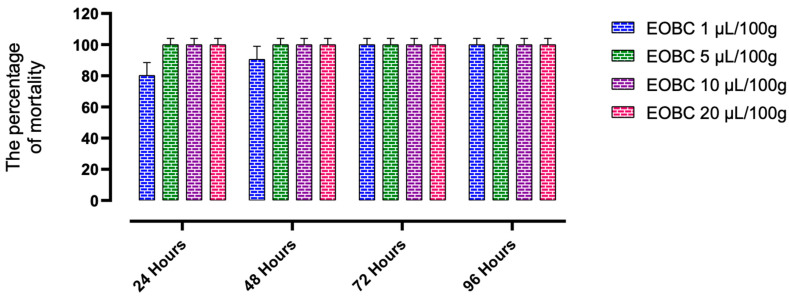
The effects of *B. cinerea* essential oil on the mortality of adults of *C. maculatus* by contact.

Figure 2 and Figure 3 present the results of the action of *B. cinerea* oils tested by inhalation and by contact on the mortality of *C. maculatus*. Indeed, at the lowest concentration (1 μL/L of air) tested by inhalation, and after 24 h of exposure, the essential oils of *B. cinerea* cause a mortality of 63.33 ± 15.28% of the adults of *C. maculatus*, while the toxicity by contact induces 80 ± 10% mortality. At the highest concentration (20 μL/100 g), *B. cinerea* EOs showed significantly higher action compared to the control and caused 100% mortality in both tests, respectively. Statistical analysis (Table 5) shows that the LC50 value in the inhalation test (0.72 μL/L of air) is higher than that observed in the contact test (0.61 μL/L of air).

Table 6 shows that the EO of *B. cinerea* (EOBC) caused a significant reduction in fecundity. Thus, the application of the low concentration (1 μL/100 g) resulted in an oviposition reduction rate of 95.98% when compared to the control group. When applying a concentration of (20 μL/100 g), the rate of egg laying reduction is 100%. The number of eggs laid per female *C. maculatus* in the control jar is 207.33 ± 12.5. For emergence, a significant reduction rate of 91.34% was observed using (20 μL/100 g).

**EOBC:** Essential oil of *B. cinerea*

**EOBC:** Essential oil of *B. cinerea*

**Table 6:** Effects of *B. cinerea* EOs on fecundity and adult emergence of *C. maculatus*.

Dosage (μm/L)	Egg Laying Reduction Rate (%)	Adult Inhibition Rate (% IR)
Control	0 ^a^	0 ^a^
1	95.9b ^a^	100 ^b^
5	98.87 ^c^	100 ^b^
10	100 ^c^	100 ^b^
20	100 ^c^	100 ^b^

All experiments were repeated 3 times. P: significant differences determined by one-way ANOVA. In each column of the table, values followed by the same letter are not significantly different from each other at *p* < 0.05 (LSD Test).

### 2.4. Cowpea Weevil Insecticidal Activity 

The results presented in Table 7 show that the *B. cinerea* EOs present a medium repulsive effect on the adults of *C. maculatus* with an average repulsion of 38.33 ± 11.38%, classifying them, respectively, in class III according to the classification of McDonald et al. [42].

Using the contact and inhalation test, the total mortality was very important; the LC_50_ was 0.62 and 0.73 µL/L, respectively. In the bibliography, several works have been conducted on the insecticidal activity of EOs or certain volatile compounds against *C. maculatus*, all using several protocols, inhalation, fumigation, contact, and repulsion.

Some EOs contain majority compounds resembling those obtained in the *B. cinerea* EOs used in the present study. Using the fumigation technique, the EOs of *Rosmarinus officinalis* L., composed mainly of α-pinene (22.64%), camphor (21.84%), 1,8-cineole (21.53%), and camphene (9.18%), show an insecticidal effect against *C. maculatus* with an LC_50_ = 15.69 µL/L air [43]. In a recent study [44], it was reported that the EOs of *Salvia officinalis*, containing α-thuyone (24.27%), camphor (18.10%), 1–8 cineol (14.38%), and β thujone (7.38%) as major components, were toxic against *C. maculatus*, while batches treated with 16 μL/L of EOs air resulted in 33.5% of eggs hatched, while 24% evolved into larvae and 19.5% was the number of emergences. It was also shown previously that EOs isolated from *Artemisia herba-alba* had an LD_50_ of 11.670% against the insect tested using the contact test, the major compounds of these oils were chrysanthenone (31.40%), camphor (15.97%), alpha-thuyone (14.90%), the 1,8-cineole (4.57%), and camphene (3.95%) [45].

EOs containing high amounts of certain volatile compounds have been used to control the insect pest *C. maculatus*. The oil extracted from *E. caryophyllus* (74.31% of eugenol), has an LC_50_ value of 1.27 μL/20 g obtained by contact test, and an LC_50_ of 20.27 μL/L air obtained by fumigation test [46]. Similarly, the EOs of *Syzygium aromaticum* L. (87.4% of eugenol) and *Cinnamomum zeylanicum* L. (73.1% of eugenol), respectively, have an LD_50_ of 78.2 and 131 μL/Kg [47]. In contrast, the use of EOs of *I. verum* (88.85% of E-anethole) against *C. maculatus* showed an LC_50_ = of 9.62 μL/20 g by contact test and an LC_50_ of 22.36 μL/L of air by fumigation test [46].

Previous studies have also reported the insecticidal effect of a few molecules against *C. maculatus.* The fumigant effect of α-bisabolol on *C. maculatus* oviposition was five eggs at a concentration of LC_50_ = 2.47 μL L^−1^ compared to 79.25 eggs at hexane application [48], while the use of citral (geranial +Neral) at a concentration of 90 μg/mL leads to a mortality of (30.00%), oviposition (55.71%), and emergence(1.51%) [49].

## 3. Materials and Methods

### 3.1. Plant Material

Aerial portions of *B. cinerea* were gathered near the town of Akka in Tata province during the second week of February 2021 (south-eastern Morocco). A botanist at the Sidi Mohamed Ben Abdellah University in Fez, Morocco, identified the plant. The laboratory of Natural Substances, Pharmacology, Environment, Modeling, Health & Quality of Life at the Faculty of Sciences Fez submitted a voucher sample (BC0019220211) at the herbarium.

### 3.2. Extraction of Essential Oils

The Clevenger hydrodistillation equipment is used to extract essential oils. For a total of 200 g of pre-dried aerial parts, together with 750 mL of water, the combination is placed in a 2-L flask and allowed to boil for three hours. Temperature was −4 degrees Celsius for the essential oils storage.

### 3.3. Analysis of the Essential Oil by GC-MS

After examining the spectrum data and retention indices of the eluted compounds, the constituents in the essential oil were identified using gas chromatography–mass spectrometry(GC/MS).The coupling was carried out using a device (GC-MS), MS of the Agilent Technologies 5973 type Brand Agilent Technologies Model 5973 with an Agilent column 19091S-433 HP-5MS, 30 m long, 0.25 mm inside diameter, and 0.25 μm film thickness of the stationary phase

Helium was employed as the carrier in the experiment, with a pressure range (psi) of 0.9 mL/s. The temperatures of the injector and detector were adjusted at 250 °C and 260 °C, respectively. The oven temperature was set between 60 and 300 degrees Celsius at a rate of 10 degrees Celsius per minute, and then kept at 300 degrees Celsius for 20 min.

### 3.4. Evaluation of Antimicrobial Activity

#### 3.4.1. Tested Strains

The antimicrobial activity of essential oils of *B. cinerea* was evaluated against 4 bacterial strains (*E. coli* (ATB: 97), *B. subtilis*, *S. aureus,* and *P. aeruginosa*) and 4 fungal strains (*C. albicans* ATCC 10231, *A. flavus*, *A. niger*, and *F. oxysporum*) which were provided, respectively, from the Laboratory of Bacteriology at the Hassan II University Hospital Center in Fez, Morocco, and the Biotechnology, Environment, Agri-Food and Health Laboratory, har El Mahraz Faculty of Sciences, Sidi Mohammed University Ben Abdellah, Fez, Morocco.

#### 3.4.2. Agar Diffusion Method

Evaluation of antibacterial and antifungal activity of *B. cinerea* essential oils were performed by agar diffusion method [50]. The four bacterial strains were grown in NB (nutrient broth) and MEA (malt extract agar) media on petri plates (*C. albicans* and inoculated with *A. niger*, *A. flavus*, and *F. oxysporum*). The inoculated medium were covered with Wattman paper discs (6 mm in diameter), which were saturated with 20 µL of *B. cinerea* essential oils. [51]. The inoculated Petri dishes were incubated at 30 °C and 37 °C in the dark for fungal and bacterial strains, respectively. Inhibition diameter and percent inhibition were determined after 24 h for the 4 bacterial strains and 48 h for *C. albicans* and after 7 days of incubation for *A. niger*, *A. flavus*, and *F. oxysporum* [51,52].

#### 3.4.3. Determination of the Minimum Inhibitory Concentration (MIC)

The determination of the MIC of *B. cinerea* EO against the 4 bacterial strains (*E. coli* (ATB:97) BGM; *S. aureus*, *B. subtilis*, and *P. aeruginosa*) and the 4 fungal strains (*F. oxysporum*, *A. niger*, *C. albicans* and *A. flavus*) was carried out by using the microdilution method in accordance with the method described by [53]. Microplates were prepared under aseptic conditions, each sterile 96-well microplate was labelled, then a volume of 100 µL of test EO in 10% (*v*/*v*) DMSO was pipetted into the first row of the plate, into all other wells, 50 µL of sterile NB for bacterial strains and 50 µL of sterile ME (malt extract) for fungal strains were added, serial dilutions were made using a multichannel pipette, and finally 30 µL of microbial suspension of each strain was added to each well. The incubation times were: 24 h for bacteria; 48 h for *C. albicans*; 7 days for fungi at 37 °C and 30 °C, respectively [53,54]. The MIC end point is determined by direct observation of growth in the wells and by using the colorimetric method using 2,3,5-triphenyltetrazolium chloride (TTC) 0.2% (*w*/*v*)) [54,55]. The evaluation of MIC, antibacterial, and antifungal activity by the Agar diffusion method, was performed in three repetitions.

### 3.5. Insecticidal Activity against C. maculatus

In this investigation, the species *C. maculatus* was investigated, which was isolated from a stock of chickpea (*Cicer arietinum*) from the Fez area. It was maintained by mass rearing carried out in glass jars of different sizes, placed in a high regulated photoperiod of 10 h of darkness and 14 h in light, a temperature of 25 °C and a relative humidity of saturation of 65 ± 5%.

#### 3.5.1. Contact Toxicity

In this assay, 100 g of seeds were infested with 5 pairs (5 male and 5 female individuals) of insects for 0–48 h, packed in sealed plastic containers with lids and covered with a smooth, transparent cloth. The EO were added to the seeds by automatic pipette and shaken manually for 2 min. The mortality of adults was evaluated after 1 day of confinement [56].

A treatment with concentration (1, 5, 10, 20 μL/ 100 g) was performed. Controls for each trial, containing 100 g of seeds (without essential oil), were also housed with 5 pairs of insects. Eggs deposited in the chickpea seeds were counted at 12 days and emerged insects were counted regularly at 28 days after confinement.

Abbott’s formula is used to correct for the observed mortality rate

$$M=100\times \frac{A-B}{100-B}$$

where M = percent corrected mortality; A = observed mortality in the tested group, and B = observed mortality in the control group.

The percent reduction in eggs and adults at each oil concentration was calculated relative to the control using the following formula

$$O=\frac{A-B}{A}\times 100$$

where O = Oviposition (or reduction of emerged insects); A = eggs number (or insects hatched in the control group), and B = eggs number (or insects hatched in the group given the EO treatment).

#### 3.5.2. Test of the Toxicity by Inhalation

Small cotton masses were hung in glass jars (1 L) by a thread linked to the inside lid. Concentrations of 1 μL, 5 μL, 10, and 20 μL/1 L of air of *B. cinerea* oil were deposited in the cotton. Ten individuals of *C. maculatus* (male and female) with ages between 0 and 48 h were placed in each jar with a tightly sealed lid. Three duplicates of each dosage were carried out. A control sample was used to make the comparison (cotton without test solutions).

The observed mortality rate is corrected by the same formula used in the contact toxicity test [57].

#### 3.5.3. Test of Repulsion 

The repellant effect of *B. cinerea* EO was tested on filter paper using the preferential area technique outlined by [42]. Thus, filter paper discs (9 cm diameter) used for this purpose were cut into two equal halves each having 31.80 cm^2^ of surface. On one of the two halves a volume of 0.5 mL of each of the essential oil solutions prepared in acetone with different doses (0.016, 0.079, 0.157, and 0.315 µL/cm^2^ per disk). Only 0.5 mL of acetone was given to the other half. The number of bruchids on the half of the disk treated with essential oil was compared to the number on the untreated half after 30 minutes.

For the calculation of the percentage of repulsion (PR), the following formula was used [58].

$$R=\frac{A-B}{A+B}\times 100$$

R = repulsion (%).

A = Insects in the control area.

B = Insects in the treatment area.

### 3.6. The Analysis of the Data

The death rate of C. maculatus was calculated using the formula of [57]. Repeated measures analysis of variance using percent mortality by toxicity was calculated for 24 h, 48 h, 72 h, and 96 h. LC_50_ concentrations were determined using the probit method [59] using “IBM SPSS Program Version 21” software.

## 4. Conclusions

Based on the obtained results, it was confirmed that the essential oils of *B. cinerea* can be used as biopesticides in the integrated management of *C. maculatus*, and it can also be used as antimicrobial alternatives to conventional antibiotics in the control of resistant strains (Fungi and bacteria). This study confirms also that the use of natural products to solve current problems is an effective and safe way to replace antibiotics and pesticides, which number of studies are providing alerts as to their upcoming problems. Further research is needed to better standardize the perfect dosage of EOs to be used for a wide range of applications.

## Figures and Tables

**Table 1:** Composition of *B. cinerea* essential oils by GC-MS.

N	RT	Scan	Type	Area	Total Height %	Total Area %	Start Time	End Time	Compound
1	4.153	17	BB	788,216.000	1.53	1.57	4.125	4.185	Camphene
2	4.398	78	BB	1,666,778.000	3.39	3.31	4.374	4.426	Sabinene
3	4.932	211	BV	451,917.632	0.91	0.90	4.908	4.952	*p*-cimene
4	4.980	223	VV	285,887.866	0.40	0.57	4.952	4.992	dl-limonene
5	5.016	232	VB	5,445,985.111	10.94	10.81	4.992	5.044	1,8 Cineole
6	5.378	322	BB	360,219.500	0.73	0.72	5.357	5.406	Trans-sabinene hydrate
7	5.647	389	BV	399,226.645	0.74	0.79	5.622	5.671	Isoamyl-2-methyl butyrate
8	5.687	399	VB	361,779.258	0.72	0.72	5.671	5.715	Cis-sabinenehydrrate
9	5.839	437	BV	1,062,072.472	2.66	2.11	5.811	5.847	2,6-Dimethyl-1,3,6-heptatriene
10	5.871	445	VB	12,539,625.462	24.23	24.90	5.847	5.895	Thujone
11	6.076	496	VB	323,419.762	0.52	0.64	6.060	6.116	Trans-chysanthemal
12	6.156	516	BV	6,822,674.056	13.55	13.55	6.128	6.181	(-)Camphor
13	6.205	528	VV	2,748,024.853	5.62	5.46	6.181	6.229	*p*-Mentha-1(7),8(10)-dien-9-ol
14	6.337	561	VB	852,987.554	1.52	1.69	6.301	6.369	(-) Borneol
15	6.426	583	BB	926,375.000	2.02	1.84	6.401	6.454	4-Terpineol
16	6.534	610	BB	811,836.000	1.67	1.61	6.510	6.562	Alpha-terpineol
17	6.819	681	BV	391,578.433	0.88	0.78	6.795	6.835	*Cis*-3-Hexenyl 2-methyl butanoate
18	7.148	763	BB	12,249,970.000	24.04	24.32	7.120	7.172	Lyratyl acetate
19	7.313	804	VB	1,059,350.468	2.30	2.10	7.293	7.341	I-Bornyl acetate
20	10.296	1547	BB	536,730.000	1.15	1.07	10.272	10.320	4-Carene
21	10.473	1591	VB	276,347.888	0.48	0.55	10.449	10.501	1,3,6-Octatriene, 3,7-dimethyl

**Table 2:** Majority compounds found in essential oils of *B. cinerea*.

Major Compound	Reference
Thujone, santolina triene, 1,8-cineol, cis-chrysanthenyl formate.	[18]
Trans-thujone, santalina triene, α- pinene, sabinene, cineole <1.8>.	[21]
α-thujone.	[22]
Iso-3thujanol; santolina triene; camphor; santolina alcohol.	[23]
Thujone; 3-carene; eucalyptol; santolina triene	[24]
(E)-citral; cis-limonene epoxide; thymol methylether; carvacrol;	[19]
Trans-thujone; cis-verbenyl acetate; 1,8-cineole; santolinatriene	[20]
Thujone; camphor; santolinatriene; eucalyptol;	[9]

**Table 3:** Antibacterial activity of *B. cinerea* EO.

	Inhibition Zone (mm)		MIC (mg/mL)	
	EO	Streptomycin	EO	Streptomycin
*P. aeruginosa*	14.66 ± 0.57	9 ± 0.5	0.0018 ± 0.0008	-
*E. coli*	26.33 ± 1.52	-	0.0037 ± 0.001	25 ± 1.63
*S. aureus*	31.33 ± 0.57	-	0.03 ± 0.012	0.62 ± 0.09
*B. subtils*	25.00 ± 0.5	10.52 ± 0.47	0.0375 ± 0.012	2.81 ± 0.095

EO: Essential oil of *B. cinerea*; (-): Resistant.

**Table 4:** Antifungal activity of *B. cinerea* EO.

	Zone Inhibition		MIC (mg/mL)	
	EO	Fluconazole	EO	Fluconazole
*C. albicans*	42.33 ± 2.08 (mm)	21 ± 1.8 (mm)	0.0168 ± 0.009	40 ± 2.29
*A. niger*	36.55 ± 2.00 (%)	89.75 ± 0.41	0.03 ± 0.012	ND
*A. flavus*	48.44 ± 2.16 (%)	94.42 ± 0.92	0.0149 ± 0.006	ND
*F. oxysporum*	88.44 ± 0.19 (%)	91.91 ± 0.9	0.06 ± 0.025	ND

EO: Essential oils of *B. cinerea*; ND: Not determined.

**Table 5:** Lethal concentration values of *B. cinerea* EOs tested on *C. maculatus*.

Treatment	DF	Slope (±SE)	LC_50_ (CI95%)	CL 95 (CI95%)	χ^2^
Inhalation	2	2.32 ± 0.34	0.72 (0.5–0.93)	3.67 (2.78–5.7)	0.71
By contact	2	3.99 ± 3.21	0.61 *	1.59 *	0.14

DF = degree of freedom; SE = standard error; CI = confidence interval; χ^2^ = Chi-deux. * Confidence intervals are too wide; they do not lend themselves to calculation.

**Table 7:** Repellent activity of *B. cinerea* EOs against insects of *C. maculatus*.

	Dose (μL/cm^2^)	Repulsion Rate (±SD)
*C. maculatus*	0.016	26.67 ± 11.54 ^a^
	0.079	33.33 ± 11.54 ^ab^
	0.157	40 ± 20 ^bc^
	0.315	53.33± 23.09 ^c^
Average PR (% ±ET)	-	38.33 ± 11.38
Repulsion class	-	Moderately repellent (III)

All experiments were repeated 3 times. P: significant differences determined by one-way ANOVA. Values followed by the same letter do not differ significantly from each other at *p* < 0.05 (LSD test).

## Data Availability

Data are available upon request.
